# Study of Adsorption Mechanism of Congo Red on Graphene Oxide/PAMAM Nanocomposite

**DOI:** 10.3390/ma11040496

**Published:** 2018-03-26

**Authors:** Mohammad Rafi, Babak Samiey, Chil-Hung Cheng

**Affiliations:** 1 Department of Chemistry, Faculty of Science, Lorestan University, Khoramabad 68137-17133, Lorestan, Iran; mohammadrafi@yahoo.com; 2 Department of Chemical Engineering, Ryerson University, Toronto, ON M5B 2K3, Canada; chilhung.cheng@ryerson.ca

**Keywords:** graphene oxide/poly(amidoamine), congo red, adsorption, KASRA model, ARIAN model, ISO equation

## Abstract

Graphene oxide/poly(amidoamine) (GO/PAMAM) nanocomposite adsorbed high quantities of congo red (CR) anionic dye in 0.1 M NaCl solution, with the maximum adsorption capacity of 198 mg·g^−1^. The kinetics and thermodynamics of adsorption were investigated to elucidate the effects of pH, temperature, shaking rate, ionic strength, and contact time. Kinetic data were analyzed by the KASRA model and the KASRA, ISO, and pore-diffusion equations. Adsorption adsorption isotherms were studied by the ARIAN model and the Henry, Langmuir, and Temkin equations. It was shown that adsorption sites of GO/PAMAM at experimental conditions were phenolic hydroxyl groups of GO sheets and terminal amine groups of PAMAM dendrimer. Analysis of kinetic data indicated that amine sites were located on the surface, and that hydroxyl sites were placed in the pores of adsorbent. CR molecules interacted with the adsorption sites via hydrogen bonds. The molecules were adsorbed firstly on the amine sites, and then on the internal hydroxyl sites. Adsorption kinetic parameters indicated that the interaction of CR to the –NH_3_^+^ sites was the rate-controlling step of adsorption of CR on this site and adsorption activation energies calculated for different parts of this step. On the other hand, kinetic parameters showed that the intraparticle diffusion was the rate-controlling step during the interaction of CR molecules to –OH sites and activation energy of this step was not calculable. Finally, the used GO/PAMAM was completely regenerated by using ethylenediamine.

## 1. Introduction

Synthetic dyes are widely used for dyeing in textile, printing, plastic, leather, paper, and cosmetic industries. These dyes exert carcinogenic effects on human health [1], and are difficult to degrade, due to their aromatic structures. For example, congo red (CR), disodium (4-amino-3-[4-[4-(1-amino-4-sulfonato-naphthalen-2-yl)diazenylphenyl]phenyl]diazenyl-naphthalene-1-sulfonate), is an anionic diazo dye [2]. In addition to its carcinogenic properties [3], which makes it highly challenging in wastewater treatment in industry, CR also shows a high solubility.

Conventional technologies, including biological degradation, coagulation, nanofiltration, ozonation, chemical oxidation, and flotation, are used with a different degree of efficiency for the removal of dyes in wastewater treatment [4,5]. Among these methods, adsorption is known as one of the most efficient methods for removing dyes from an aquatic environment. Hence, an adsorbent with a high adsorption capacity of synthetic dyes is highly sought.

Many compounds have been used to absorb CR from aqueous solutions, such as acid activated red mud [6], coir pith carbon [7], bentonite [8], bohemite [9], eggshell membrane [10], Ca-bentonite [11], carbon nanotubes [12], polyhedral Cu_2_O [13], TiO_2_-graphene [14], graphene oxide/chitosan [15], and filter paper [16].

In this work, graphene oxide/poly(amidoamine) nanocomposite was used as an adsorbent, due to its high adsorption capacity for dyes and metal ions [17,18]. This nanocomposite was synthesized by functionalizing graphene oxide (GO) with poly(amidoamine) (PAMAM) dendrimer through a grafting to method [19].

GO is made of graphite oxide. Graphite oxide is prepared by treating graphite with strong oxidants. The resulted graphite oxide has various ratios of carbonyl, carboxyl, hydroxyl, phenol, and epoxy groups, depending on the synthesis method and oxidation degree [20]. The layer structure of graphite oxide shows more irregular and larger spacing than graphite. Due to its distinct plate structure, superb stability, relatively large specific surface area, and rich functionalities, GO has a wide range of applications in medicine, chemistry, physics and biology, biosensing, and drug delivery [21,22,23,24,25].

On the other hand, PAMAM is a class of dendrimer that is synthesized by repetitively branching subunits of amide and amine functional groups. Its internal molecular structure consists of tree-like branching, and each external layer (or generation) contains exponentially more branching points, which results in numerous surface sites relative to the total molecular volume [26,27]. Furthermore, due to the relatively low cost of synthesis and their functionalizability, PAMAM dendrimers have been recognized as an appropriate material for adsorption applications [28].

In this work, the synthesized GO/PAMAM nanocomposite was characterized by various techniques, such as XRD (X-ray diffraction), FTIR (Fourier-transform infrared), SEM (scanning electron microscope), BET (Brunauer-Emmett-Teller), and Zeta potential measurement. The CR adsorption capacity of GO/PAMAM was evaluated using various experimental variables, for instance, temperature, pH, contact time, shaking rate, ionic strength, and concentration of CR. In order to identify the adsorption mechanism, analyses of thermodynamics and kinetics of the adsorption process were calculated using the ARIAN and KASRA models, respectively.

## 2. Experimental

### 2.1. Chemicals

Graphite powder (<20 nm) was purchased from Sigma-Aldrich (St. Louis, MO, USA). Congo red, orange G, sodium nitrate, potassium permanganate, sodium hydroxide, sodium chloride, concentrated sulfuric acid (98%), hydrochloric acid (37%), hydrogen peroxide (30%), methanol (≥99.9%), ethanol (≥99.9%), ethylenediamine (≥99%), methyl acrylate (≥99%), *N*,*N*-dimethy etherlformamide (DMF) (≥99.8%), tetrahydrofuran (≥99%), diethylenetriamine (≥98%), acetone (99.8%), diethyl ether (≥99.7%) and carbon tetrachloride (99.5%) were purchased from Merck (Darmstadt, Germany). All of the chemicals were used without further purification.

### 2.2. Synthesis of PAMAM

In this work, Generation 2 PAMAM (G2 PAMAM) dendrimer was synthesized from methyl acrylate and ethylenediamine, using pubished procedures [26,27].

### 2.3. Synthesis of Graphene Oxide (GO)

GO was synthesized using a modified Hummer’s method [29,30]. Briefly, 0.2 g of graphite powder and 0.2 g of NaNO_3_ and 9 mL of concentrated sulfuric acid were added into a 250 mL round bottom flask and were stirred at 600 rpm for 1 h at room temperature. Then, the flask was immersed in an ice bath, followed by the gradual addition of 1.2 g of KMnO_4_, whereas the dark green reaction mixture was stirred at 600 rpm. After 2 h, 18.4 mL of distilled water was added dropwise into the solution and its color turned dark brown. Afterwards, the flask was immersed in an oil bath at 98 °C for 30 min. To this mixture, 40 mL of distilled water and 4 mL of H_2_O_2_ (30%) were added, and was kept stirring at 98 °C for 15 min. In all of the above steps, the reaction mixture was stirred continuously at 600 rpm. Then, the reaction mixture was filtered and washed six times with 10% HCl. Later, distilled water was added to the filtrate and each time was centrifuged at 6000 rpm until the water became neutral. Finally, by dispersing graphite oxide in DMF and sonicating for 0.5 h, graphene oxide was prepared.

### 2.4. Synthesis of GO/PAMAM

The GO/PAMAM was prepared according to the published procedure [16,31]. A solution of 5 g PAMAM dissolved in 20 mL of methanol was added dropwise to a round-bottomed flask, in which 120 mL of DMF solution containing 1 g exfoliated GO, was stirring. Subsequently, the solution was refluxed at 80 °C for 24 h. After this step, the warm solution was filtered and washed with 200 mL ethanol. The resultant product was then dispersed in 200 mL ethanol by mechanical agitation four times until no precipitation was observed. The product was transferred into a glass dish and was dried under vacuum at 100 °C for 12 h.

### 2.5. Characterization of GO/PAMAM

**Figure 1:**
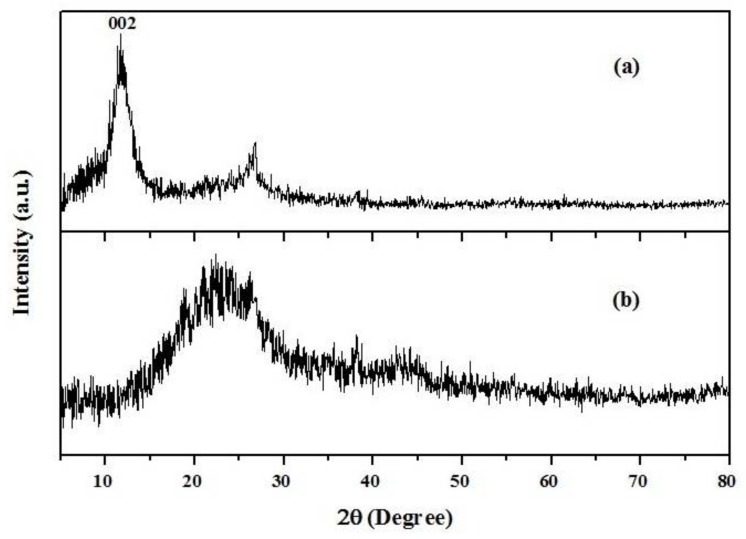
XRD (X-ray diffraction) spectra of (**a**) graphene oxide (GO) and (**b**) graphene oxide/poly(amidoamine) (GO/PAMAM).

The crystal structure of GO/PAMAM was recorded by a Rigaku D-max C III, X-ray diffractometer (Rigaku Corporation, Tokyo, Japan) using Ni-filtered Cu-Kα radiation (*λ* = 1.5406 Å). As seen in Figure 1a, the diffractogram of GO showed a sharp peak at 11.7° corresponding to interlayer distances (d_002_) of 0.756 nm and a peak at 26.6°. However, the GO diffraction peak disappeared in the XRD pattern of GO/PAMAM nanocomposite, showing an excellent exfoliation and dispersion of GO in the produced GO/PAMAM, Figure 1b. Also, in the XRD pattern of GO/PAMAM, a broad peak was observed at 26.7° [32,33], which indicated its amorphous nature.

**Figure 2:**
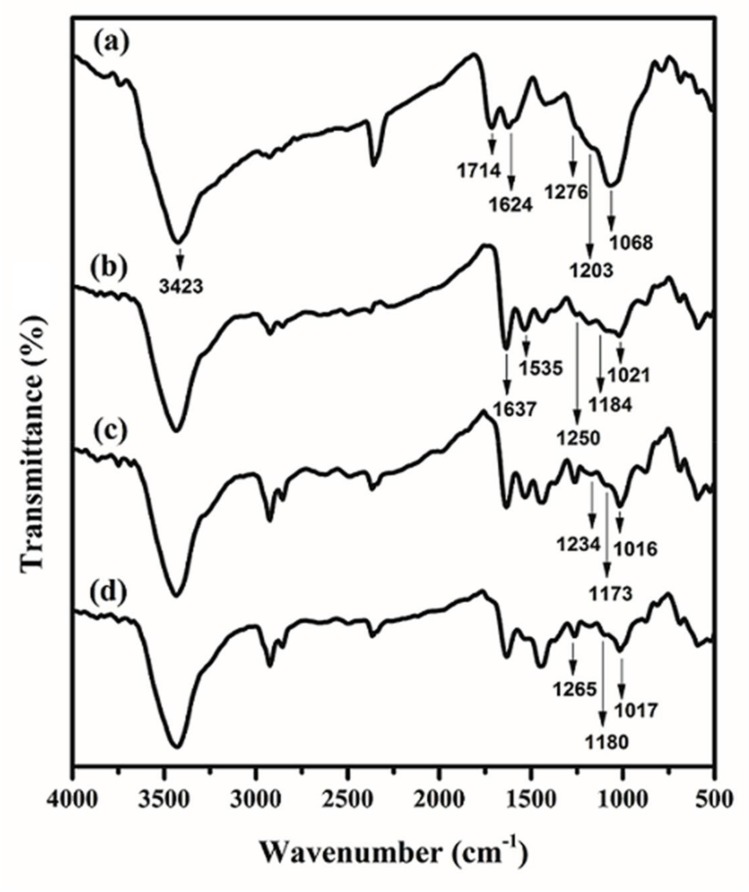
FTIR spectra of (a) GO; (b) GO/PAMAM; (c) congo red (CR)-adsorbed GO/PAMAM; and, (d) GO/PAMAM at pH = 12.

IR spectra of GO, PAMAM, and GO/PAMAM were attained with a Nicolet IR 100 (Thermo Scientific, Waltham, MA, USA) FTIR spectrophotometer using KBr pellets, Figure 2. The peaks at 1714, 1624, 1276, 1068 and 3423 cm^−1^ in IR spectrum of GO were assigned to the stretching vibrations of C=O bond of carbonyl or carboxyl, C=C, C−O−C, C−O, and −OH groups of GO [34], respectively, Figure 2a. In the GO/PAMAM IR spectrum, the C=O peak of GO (1731 cm^−1^) completely diminished, which was assigned to its interaction with amine groups of PAMAM, as shown in Figure 2b. The appearance of the peaks at 1637 cm^−1^ (C=O amide I stretching) and 1535 cm^−1^ (−CONH−) [16,34] in GO/PAMAM spectrum confirmed that GO/PAMAM was successfully synthesized.

**Figure 3:**
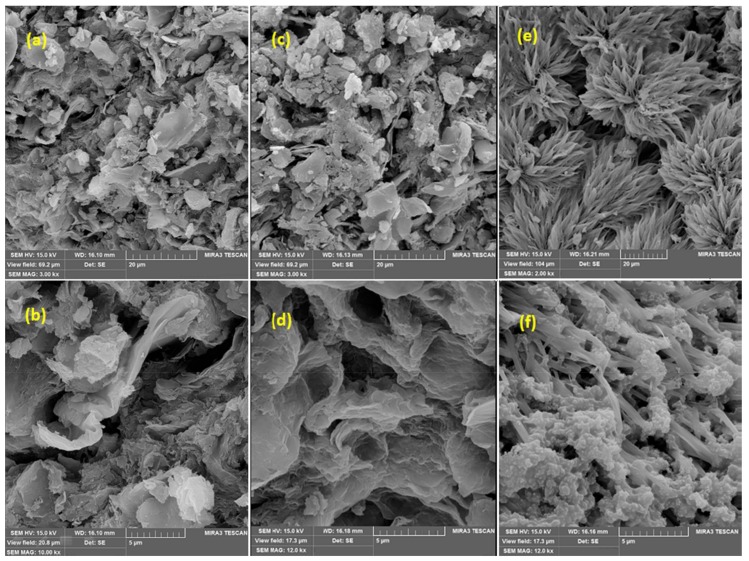
SEM images of GO/PAMAM at magnifications of (**a**) ×3k and (**b**) ×10k; CR-adsorbed GO/PAMAM at magnifications of (**c**) ×3k and (**d**) ×12k and GO/PAMAM at pH = 12 at magnifications of (**e**) ×2k and (**f**) ×12k.

Scanning electron micrographs of GO/PAMAM, CR-adsorbed GO/PAMAM and GO/PAMAM at pH of 12 were taken using a MIRA3 TESCAN instrument (Kohoutovice, Czech Republic) at 15 keV, Figure 3. SEM photo showed that GO/PAMAM nanocomposite was an agglomeration of platelike particles and its surface was uneven, Figure 3a,b. A similar morphology was observed for CR-adsorbed GO/PAMAM in Figure 3c,d. However, Figure 3e,f show a lotus flower-like morphology for GO/PAMAM at pH = 12, possibly due to the neutralization of phenolic –OH and –NH_3_^+^ groups of adsorbent at the alkaline environment. This showed that GO sheets and their phenolic –OH groups were located in the internal surface (pores) of adsorbent and repulsion interaction between them after neutralization changed the morphology of adsorbent.

Zeta potential of GO/PAMAM was measured as −49.5 mV, using a Malvern (Zetasizer-nono zs, Malvern Pananalytical Ltd., Malvern, UK) zeta potential meter. This implies that there were lots of carboxylate groups on the GO sheets of adsorbent. However, Zeta potential measurements of CR-adsorbed GO/PAMAM and GO/PAMAM at pH = 12 were not successful, due to their poor dispersity in water. This observation was rationalized, as follows: at pH of 12, C–O^−^ groups resulted from the neutralization of phenolic −OH groups of GO can interact with other groups like –NH_2_ groups of PAMAM. In addition, as CR molecules were adsorbed on the adsorbent surface, interactions of two sulfonate groups of CR molecule with GO/PAMAM surface resulted in the formation of aggregations of adsorbent particles and prevented their dispersion. This confirmed the results that were obtained from SEM images.

**Figure 4:**
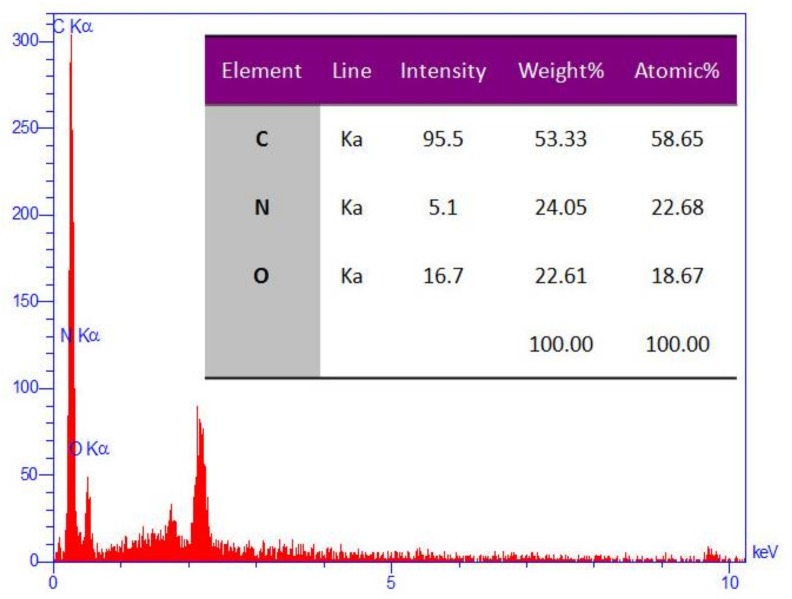
The EDS (Energy-dispersive X-ray spectroscopy) analysis of GO/PAMAM.

EDS (Energy-dispersive X-ray spectroscopy) analysis of synthesized nanocomposite (by a MIRA3 TESCAN instrument) showed that atomic nitrogen percentage of its surface was 23% of total atomic percentage that verified the formation of GO/PAMAM, Figure 4.

The nitrogen-based BET specific surface area of GO/PAMAM was achieved by a Micrometrics-Tristar 3020 equipment (Micrometrics, Narcross, GA, USA), Figure S1. The resulted isotherm is type IV. These isotherms are the characteristic of porous materials and nitrogen molecules were condensed in the tiny capillary mesopores of adsorbent. The BET surface area, t-plot micropore area, adsorption average pore diameter (by BET), and pore volume for GO/PAMAM were 3.26 m^2^·g^−1^, 1.19 m^2^·g^−1^, 18.0 nm, and 0.015 cm^3^·g^−1^, respectively. The hysteresis loop of BET isotherm was H3, which was originated from aggregates (loose assemblages) of platelike particles (here GO sheets of adsorbent) forming slit-like pores [35]. This arrangement was supported by SEM images of adsorbent and could be responsible for broad peak observed at 26.7° in diffractogram of GO/PAMAM.

### 2.6. Adsorption Studies

#### 2.6.1. Adsorption Experiments

The adsorption experiments were carried out in a series of 15-mL glass bottles. 0.002 g of GO/PAMAM as the adsorbent was added to each bottle, followed by charging a volume of 10 mL CR solution with a predetermined initial concentration in it. The solutions were shaken at 100 rpm in a temperature controlled shaking water bath (Fater electronic Co. (Tehran, Iran), Persian Gulf model) at 308, 318, and 328 K within ±0.1 K for 10 h to reach equilibrium under experimental conditions. The initial concentration ranges of CR were 3 × 10^−6^–8.5 × 10^−5^ M. After adsorption, the residual concentrations of CR were determined by photometry (UV mini 1240 V, Shimadzu (Kyoto, Japan)) at their $\lambda_{\max}$ values in these solutions. The $\lambda_{\max}$ value of CR in water was 489 nm. The adsorption capacity of CR on the adsorbent, $q_{e}$ (mg·g^−1^), was calculated as follows

(1) $$q_{e}=\frac{\left(c_{0}-c_{e}\right)Mv}{1000w}$$

where $c_{0}$ and $c_{e}$ are the initial and equilibrium (or residual) concentrations of adsorbate (M), respectively, *v* is the volume of solution (mL), *w* is the weight of the used adsorbent (g), and *M* is the molecular weight of adsorbate (mg·mole^−1^). In adsorption kinetic experiments, to a series of glass bottles, each bottle was charged with a 10 mL of CR aqueous solutions with an initial concentration of 2 × 10^−5^, 5 × 10^−5^, or 9 × 10^−5^ M and 0.002 g of GO/PAMAM. The solutions were shaken at 40, 70, and 100 rpm and different temperatures. At designated contact times, the concentrations of CR in the solutions were determined by photometry at their $\lambda_{\max}$ values. In these series of experiments, $q_{e}$ and $c_{e}$ in Equation (1) were replaced by $q_{t}$ (adsorption capacity at time *t*) and $c_{t}$ (concentration of adsorbate at time *t*), respectively.

#### 2.6.2. Adsorption Thermodynamic Models

The adsorption isotherms were studied by “*adsorption isotherm regional analysis Model*” (abbreviated as ARIAN model) [36,37]. ARIAN is a Persian word meaning Iranian. This model has been introduced for studying adsorption isotherms up to four regions. In the ARIAN model, which is explained briefly, it is assumed that region I obeys the Henry’s law:

(2) $$q_{e}=Kc_{e}$$

where *K* is the binding constant of adsorbate on the surface and adsorption increases linearly with concentration. Region II starts from the *starting second region concentration* (abbreviated as *ssc*) point. In this region occurs only when a monolayer surface aggregate forms and can be studied by an appropriate isotherm such as the Langmuir, Temkin, equations, and etc. The Langmuir equation [38] in linearized form is represented as

(3) $$\frac{c_{e}}{q_{e}}=\frac{1}{q_{\max}K}+\frac{c_{e}}{q_{\max}}$$

where *K* is the Langmuir adsorption equilibrium constant and $q_{\max}$ is the monolayer capacity of adsorbent. The Temkin equation [39] is given by

(4) $$q_{e}=c_{1}\ln\left(c_{2}c_{e}\right)$$

where $c_{1}$ is a constant and $c_{2}$ is the adsorption equilibrium constant.

**Figure 5:**
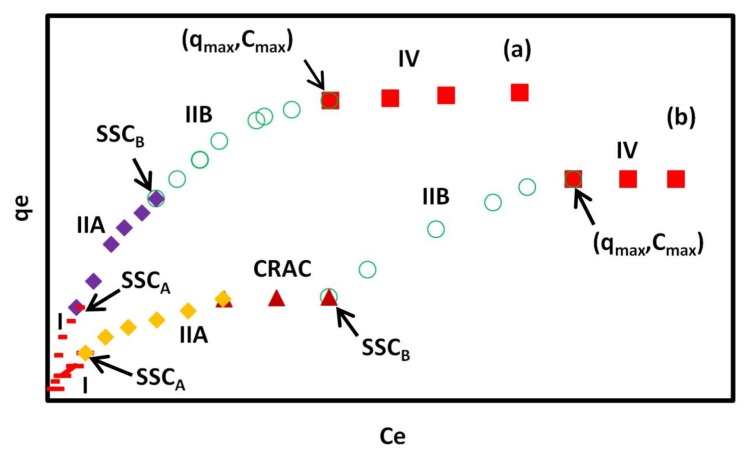
Typical adsorption isotherms of CR on GO/PAMAM nanocomposite in (a) water and (b) alkaline solutions according to the ARIAN model. Symbols –, ♦, ○, ■ and ▲ show I, IIA, IIB, and IV regions and CRAC, respectively. CRAC is an abbreviation for “*concentration range of leveling off between two successive adsorption isotherm curves*”.

In region III, new surface aggregates of molecules (or admicelles) and new surface clusters (in the case of surfactants) form. The *starting third region concentration* (abbreviated as *stc*) point defines the beginning of this region. These data are analyzed by the bilayer isotherm equation, Equation (5), and the equations that are derived from it, Equations (6) and (7) [37]. In region III, by assuming that adsorption occurs mostly in the first and second layer, we have

(5) $$\frac{c_{e}}{q_{e}}=\frac{1+c_{e}K_{sa}}{q_{mon}K_{sa}}+\frac{xc_{e}^{2}K_{sa}}{2q_{mon}xc_{e}K_{sa}}$$

where $q_{mon}$ and $q_{e}$ are the monolayer and equilibrium adsorption capacity, respectively $K_{sa}$ and *x* are the adsorption equilibrium constants of adsorbate molecules in the first layer surface aggregates, and, that of adsorbate molecules in all of the layers excluding the first layer, respectively. If adsorbate molecules are adsorbed mostly on the first layer, Equation (5) can be re-written as

(6) $$\frac{c_{e}}{q_{e}}=\frac{1}{q_{mon}K_{sa}}+\frac{c_{e}}{q_{mon}}+\frac{xc_{e}^{2}}{q_{mon}}$$

which is used for *low bilayer coverage* (abbreviated as LBC) and if the adsorption process yields a monolayer formation, Equation (5) can be further reduced to

(7) $$\frac{c_{e}}{q_{e}}=\frac{1}{q_{mon}K_{sa}}+\frac{c_{e}}{q_{mon}}$$

where Equation (7) is a Langmuir-type equation. The region IV starts where the adsorption capacity reaches the maximum, showing a plateau in the isotherm, or where the isotherm begins to goes down. The second situation in region IV is called the reverse desorption and obeys from the reverse desorption equation [37]. Schematic adsorption isotherms of CR on GO/PAMAM, according to the ARIAN model, are shown in Figure 5.

#### 2.6.3. Adsorption Kinetic Models

The kinetic data were analyzed by a number of equations. The pore-diffusion equation [40] is given as:

(8) $$q_{t}=k_{dif}t^{0.5}+I$$

where *I* is proportional to the boundary layer thickness and $k_{dif}$ is the rate constant for intraparticle diffusion.

Also, for an analysis of adsorption kinetics, the KASRA model and KASRA equation [41,42] were used. KASRA is abbreviated from “*kinetics of adsorption study in the regions with constant adsorption acceleration*” and is a synonym of “king” in Persian. The KASRA model is based on the following assumptions: (1) each time range that adsorption acceleration in it is constant, is named a “*region*”; (2) there are two regions before reaching the plateau region; and, (3) the boundaries between the first and second regions and the second and third (plateau) regions are named *starting second region* *(abbreviated as ssr) and kinetics of adsorption termination* (abbreviated as *kat*) points, respectively. Both *ssr* and *kat* points are determined by the KASRA equation [42], shown as follows:

(9) $$q_{t}=\frac{1}{2}a_{i}t^{2}+\left(v_{0i}-a_{i}t_{0i}\right)t+q_{0i}-\frac{1}{2}a_{i}t_{0i}^{2}-\left(v_{0i}-a_{i}t_{0i}\right)t_{0i}$$

where $q_{0i}$, $v_{0i}$, and $t_{0i}$ are $q_{t}$, velocity and time values at the beginning the *ith* region, respectively, $a_{i}$ is the acceleration of adsorption kinetics in the *ith* region, whereas *i* = 1–3. Each $a_{i}$ is a negative value because of the decrease in the adsorbate concentration during the adsorption process. In the first region, $t_{01}$ and $q_{01}$ values are equal to zero. The second region starts from the *ssr* point, which is assigned with the coordinates $q_{02}$ and $t_{02}$. An alternative format of the KASRA equation is represented as:

(10) $$q_{t}=At^{2}+Bt+C$$

where $A=\frac{1}{2}a_{i}$, $B=v_{0i}-a_{i}t_{0i}$ and $C=q_{0i}-\frac{1}{2}a_{i}t_{0i}^{2}-\left(v_{0i}-a_{i}t_{0i}\right)t_{0i}$. Finally, the plateau (third) region begins at the equilibrium time, $t_{e}$ and equilibrium adsorption capacity $q_{e}$, which are coordinates of *kat* point. In this region, $v_{03}=a_{3}=0$, $q_{03}=q_{e}$ $t_{03}=t_{e}$ and Equation (10) is simplified to $q_{t}=q_{e}$. Due to different features of the first and second regions, parameters in these two regions, such as rate constants that are obtained from the initial adsorption rate, and etc. are different from each other, and the related equations for these regions come different pathways from the point $q_{t}=0$ at t=0.

In the KASRA model, the number of regions is written in two forms. For example, the first region can be written as region 2. In this work, to avoid confusion in relation to the regions in isotherms and kinetic curves, kinetic regions are shown using numbers, like region 1 and etc.

**Figure 6:**
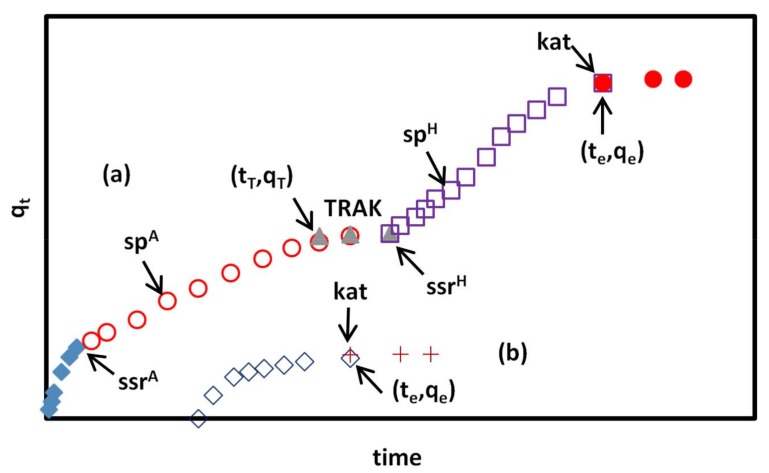
(a) Typical bi-curve adsorption kinetic diagram of CR adsorption on GO/PAMAM in water and 0.1 M NaCl solutions according to the KASRA model. The first bi-regional kinetic curve ends at $\left(t_{T},\;q_{T}\right)$ point and the second single-region kinetic curve starts from $ssr^{H}$ point. The superscripts of *A*, *H*, and *NH* refer to –NH_3_^+^, hydroxyl, and –NH_2_ sites, respectively. In alkaline solutions (except at pH = 13), the superscript H is replaced by *NH*; (b) At pH of 13, the diagram composes of a single-region curve, including only –NH_2_ sites. $sp^{A},\;sp^{H}$ and $sp^{NH2}$ symbols refer to *sp* points in the ISO equation. TRAK is an abbreviation for the “*time range of interval between two successive adsorption kinetic curves*”.

Schematic adsorption kinetic curves of CR on GO/PAMAM, according to the KASRA model, were shown in Figure 6.

The *ideal-second-order* (or abbreviated as ISO) equation [43] is shown as

(11) $$\ln\left(\frac{q_{e}-q_{t}}{ac_{t}}\right)=-\frac{k_{I}c_{e}}{q_{e}}t+A^{\prime }$$

where $k_{I}=k_{I}^{2}q_{e}$ and $k_{I}^{2}$ are the first- and second-order adsorption rate constants of the ISO equation in each region and are in M^−1^ mg·g^−1^ min^−1^ and M^−1^ min^−1^, respectively and $A^{\prime }=\ln\left(\frac{q_{e}}{ac_{0}}\right)$, $a=\frac{Mv}{1000w}$, where *v* is the volume of solution (mL), *w* is the weight of the used adsorbent (g) and *M* is the molecular weight of adsorbate (mg·mole^−^^1^). Some adsorbents have *m* different adsorption sites and adsorption occurs on the first, then second,…, (*m*−1)*th* and *mth* sites respectively. In these cases, in Equation 11 $q_{e}$ and $c_{e}$ are used for *mth* site and for *m*−1 other sites are replaced with $q_{t,max}^{i}$ and $c_{t,max}^{i}$, where *i* = 1,… *m*−1. $q_{t,max}^{i}$ and $c_{t,max}^{i}$ are the maximum adsorption capacity of adsorbent and adsorbate concentration after absorption completion on the *ith* adsorption site, respectively.

As referred to before, based on the KASRA model, there are two regions in adsorption kinetic curves before reaching the plateau, which results from non-ideality in adsorption. In the first one, completely ideal adsorption occurs on the bare surface of adsorbent. The progressively changes occurred on the surface of adsorbent in region 1 finally results in emerging another ideal region (region 2), in which the adsorption is carried out on a partly adsorbate-covered surface. Using the ISO equation shows that region 2 is composed of two another ideal parts, which are named 2a and 2b. The first part of the second region, 2a, starts after *ssr* point and the second one, 2b, starts after *starting second part* (or abbreviated as *sp*) point and ends at the *kat* point.

The ISO second-order rate constant of region 1 is shown with $k_{I1}^{2}$ and and those of the second region are shown with $k_{I2a}^{2}$ and $k_{I2b}^{2}$. As referred, in some adsorbents, there are two or more different adsorption sites that result in observing two or more successive adsorption kinetic curves in adsorption kinetic diagram. In these cases, region 1 (completely ideal) is only observed in the first adsorption kinetic curve, Figure 6.

Sometimes, due to braking effect [43], an interval is observed between two successive adsorption kinetic curves of two different sites or between regions 1 and 2 of the first adsorption curve. The “*time range of interval between two successive adsorption kinetic curves*” (abbreviated as TRAK) is used to compare this effect in different cases. If adsorption results in a TRAK, $q_{t,max}^{i}$ and $c_{t,max}^{i}$ are replaced by $q_{T}^{n}$ and $c_{T}^{n}$. $q_{T}^{n}$ and $c_{T}^{n}$ are adsorption capacity of adsorbent and adsorbate concentration at the beginning of the TRAK between *nth* and (*n*+1)th kinetic curves, respectively, and in these cases, $k_{I}=k_{I}^{2}q_{T}^{n}$.

## 3. Results and Discussion

### 3.1. Adsorption Isotherm Modeling

**Figure 7:**
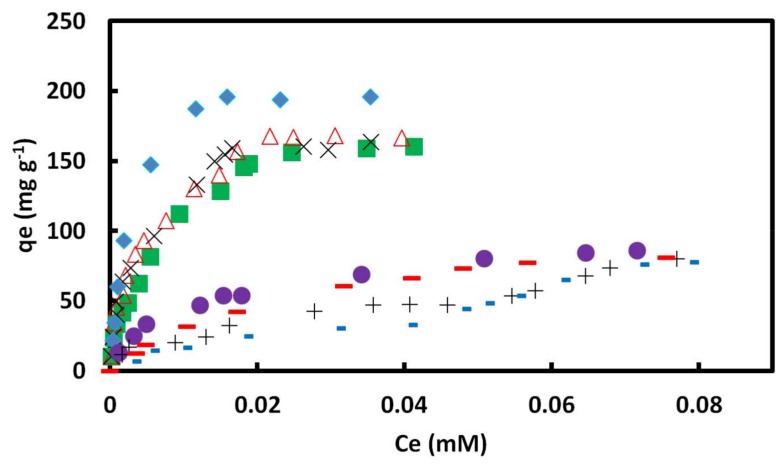
$q_{e}$ versus $c_{e}$ for adsorption of CR on GO/PAMAM nanocompositefrom 0.05 mM CR at ■ 308, ∆ 318 and ×328 K in water and in ♦ 0.1 M NaCl, ● pH = 10, + pH = 11, − pH = 12 and ▬ pH = 13 solutions at 318 K.

Studying adsorption isotherms is an important tool to unveil the mechanism of an adsorption system. In this work, a second generation (G2) of GO/PAMAM nanocomposite was used as the adsorbent. The adsorption capacity ($q_{e}$) of CR on the GO/PAMAM in the initial CR concentration range of 3 × 10^−6^–8.5 × 10^−5^ M in aqueous solutions at 308, 318, and 328 K are shown in Figure 7. The maximum adsorption capacity ($q_{e,\max}$) values of the process were 155.9, 167.6, and 160.2 mg·g^−1^ at 308, 318, and 328 K, respectively. The relatively high $q_{e}$ values of this adsorption process are attributed to the porous structure of GO/PAMAM nanocomposite [32].

Analysis of equilibrium adsorption data by the ARIAN model showed that in the used concentration range of CR, these adsorption isotherms were composed of regions I and II (region II includes sections IIA and IIB), before attaining the plateau (region IV). Similar behaviors were seen in the adsorption isotherms of acid Bordeaux B on GO/PAMAM [17]. Binding constants of adsorption process in region I were calculated by Henry’s law, Table 1, and ∆H and ∆S values of adsorption calculated using them in this region were 17.5 kJ·mol^−1^ and 203.5 J·mol^−1^·K^−1^, respectively. By fitting the rest of data using equations proposed by the ARIAN model, Equations (3)–(7), it was observed that region II was composed of two sections. These sections were called IIA and IIB, and their related parameters were indexed by A and B letters. The indices show that there are two different kinds of adsorption sites on the surface of GO/PAMAM. It was observed the data fitting in region II was better using the Temkin equation than using the Langmuir equation. The binding constants obtained from the Temkin equation are shown in Table 2 and Table 3, and were used to calculate related thermodynamic parameters of sections IIA and IIB. ∆H and ∆S values of the adsorption process in section IIA were 7.4 kJ·mol^−1^ and 153.4 J·mol^−1^·K^−1^, and those of section IIB were 23.0 kJ·mol^−1^ and 188.8 J·mol^−1^·K^−1^, respectively.

**Figure 8:**
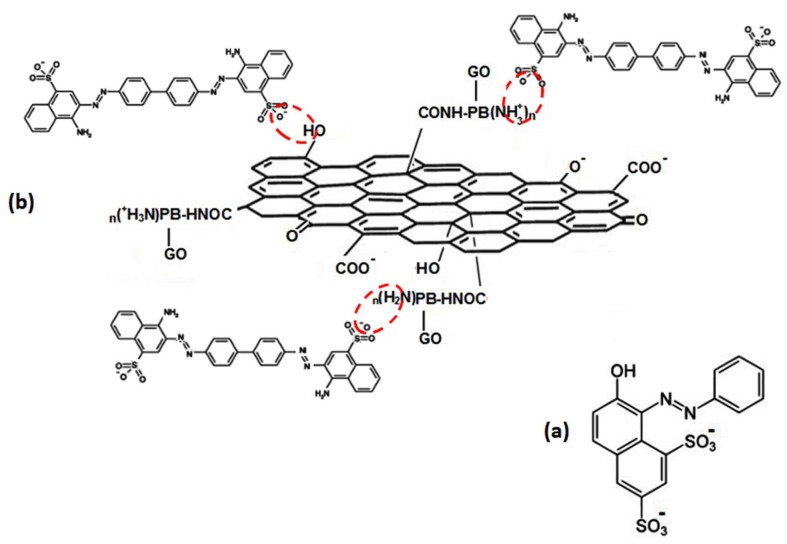
(**a**) Orange G and (**b**) adsorption sites of GO/PAMAM to CR. PB is an abbreviation for PAMAM BODY.

Hydrophobic interactions between GO sheets [44] and PAMAM dendrimer of the GO/PAMAM to CR molecules play a key role in the observed endothermic interactions [45]. To verify the role of hydrophobic interactions in the adsorption of CR on GO/PAMAM, we examined the adsorption of orange G dye on GO/PAMAM. Orange G is a doubly–negative charged dye that is similar to CR, but it is about half the size of CR molecule, Figure 8a. Our tests showed that orange G was not adsorbed on GO/PAMAM. Also, we synthesized GO–DETA (graphene oxide-diethylenetriamine) [46] and examined its adsorption capacity of CR molecules. CR molecules were not adsorbed on GO–DETA. These observations showed the role of hydrophobic interactions between the hydrocarbon moiety of CR molecule and GO/PAMAM surface. As seen in Table 4, the relative magnitudes of the region I and sections IIA and IIB were proportional to $\frac{SCC_{A}}{q_{e,\max}}$, $\frac{SCC_{B}-SCC_{A}}{q_{e,\max}}$ and $\frac{q_{e,\max}-SSC_{B}}{q_{e,\max}}$, respectively. As the temperature increased, the relative magnitude of region I was fairly constant, and the relative magnitudes of sections IIA and IIB were increasing and decreasing correspondingly.

CR has negatively charged sulfonate groups that can interact with polar groups of the GO/PAMAM nanocomposite. As reported before [34], the start points of deprotonation of carboxyl and phenolic –OH groups of GO sheets and –NH_2_ (primary amine) groups of PAMAM were at pHs of 4.76, 8.24, and 10.51, respectively. Zeta potential of GO/PAMAM was −49.5 mV, which indicated that carboxylic acid groups of GO sheets of adsorbent in neutral water were as carboxylate groups. Thus, the carboxylic acid groups of adsorbent were not considered as the adsorption sites for CR. As researchers reported, GO adsorbed CR molecules by its phenolic –OH groups [47] and polypyrrole and polyaniline adsorbed CR by their amine groups [48].

Thus, –OH groups of GO sheets and the protonated form of –NH_2_ (–NH_3_^+^) groups of PAMAM were the adsorption sites of GO/PAMAM in neutral solutions (or as we call “water” in this work), and as was observed –NH_2_ group of PAMAM and its protonated form (–NH_3_^+^) were adsorption sites of GO/PAMAM in alkaline solutions, Figure 8b.

According to the EDS analysis in Figure 4, the atomic percentages of N and O atoms of the adsorbent surface were 22.7% and 18.7%, respectively. By considering the PAMAM structure and its amide bondings to the surface of GO sheets of adsorbent, a considerable part (more than 12%) of these surface O atoms belonged to PAMAM. On the other hand, BET surface of GO/PAMAM synthesized in this work was 3.26 m^2^·g^−1^ when compared to BET surface value (180 m^2^·g^−1^) reported for graphene oxide prepared by dispersion [49]. These observations indicated that most of GO sheets were covered by PAMAM, and therefore most –OH groups of GO sheets were located in the adsorbent pores.

At the beginning, charged terminal –NH_3_^+^ groups of PAMAM interact with CR molecules, which act as the adsorption sites in region I and section IIA. The adsorption sites in region I and section IIA are the same type, but the former are more active than the latter ones. The –OH groups of GO sheets are the adsorption sites in section IIB. As given in Table 1, Table 2 and Table 3, the binding constants of CR molecules to the GO/PAMAM decrease from region I to section IIB. Due to the progressively increasing negative charge of CR–adsorbed GO sheets and the spatial hindrance of these adsorbed CR molecules, the binding constants of adsorption process in region I are greater than those in section IIA. Furthermore, the peripheral –OH groups of GO sheets are less polar than –NH_3_^+^ groups of adsorbent. It makes the interaction of CR molecules with –NH_3_^+^ groups of adsorbent (in section IIA) stronger than its interaction with –OH ones in section IIB. As seen in Figure 2a, the stretching mode of C–O (phenolic) group in the spectrum of GO/PAMAM appeared at 1203 cm^−1^ [50]. In Figure 2b,c, the peaks of stretching modes of C–O (phenolic) group at 1184 cm^−1^ and C–N (primary aliphatic amine) group at 1250 cm^−1^ in the IR spectrum of GO/PAMAM shifted to1173 and 1234 cm^−1^ in the IR spectrum of CR–adsorbed GO/PAMAM, respectively. These red shifts were due to the formation of hydrogen bonds between these groups and sulfonate groups of CR molecules.

#### Effect of pH and Ionic Strength on the Adsorption of CR on GO/PAMAM

As reported [32], the point of zero charge (pzc) of GO/PAMAM, i.e., the point at which the surface charge is neutral, was 7. The number of phenolic –OH groups on GO sheets decreased with the increase of pH. All of the phenolic –OH groups were deprotonated at pH ≥ 8.24 and the –NH_3_^+^ groups started to be deprotonated at pH ≥ 10.51 [34].

Due to the neutralization of phenolic –OH and –NH_3_^+^ groups of GO/PAMAM, the stretching modes of C–O (phenolic) group at 1184 cm^−1^ and C–N group at 1250 cm^−1^ in the IR spectrum of GO/PAMAM shifted to 1180 and 1265 cm^−1^ in the IR spectrum of GO/PAMAM at pH = 12, respectively, Figure 2b,d. In high pH environment, only –NH_2_ and –NH_3_^+^ groups were capable of adsorbing CR molecules. Due to the lack of –OH groups, adsorption isotherms obtained at alkaline pHs showed that $q_{e,\max}$ of GO/PAMAM decreased considerably at pHs of 10, 11, 12 and 13. At pHs of 10, 11 and 12, CR molecules were adsorbed on –NH_3_^+^ sites in region I and section IIA, followed by adsorbing on –NH_2_ sites in section IIB. As it was observed from $q_{e,\max}$ values of sections IIA and IIB, the number of –NH_3_^+^ sites decreased and that of –NH_2_ sites increased with increase of pH. At pH of 13, only –NH_2_ sites existed on the surface of adsorbent. Under this circumstance, the isotherm was composed of region I and a single section region II, Figure 7. As reported [51], for the adsorption of methylene blue on reduced graphene oxide/poly(acrylamide) nanocomposite, only the poly(acrylamide) chains of nanocomposite were capable of adsorbing methylene blue molecules, because no hydroxyl groups appears on the reduced graphene oxide sheets.

The $q_{e,\max}$ values of all the isotherms in alkaline solutions were very similar and in the range of 80–86 mg·g^−1^, which showed the total number of –NH_2_ and –NH_3_^+^ groups on the adsorbent surface. Analysis of these adsorption isotherms by the Henry, Temkin, and Langmuir equations showed that, in region I and sections IIA and IIB, the binding constants of CR molecules to the surface decreased with the increase in pH from 10 to 12, Table 1, Table 2 and Table 3. This resulted from the neutralization of–NH_3_^+^ groups, which increased the negative charge of GO/PAMAM surface and therefore the repulsion interaction between the CR molecules and adsorbent. However, it is also noticeable that an increase in the binding constants of different regions at pH of 12, and a small increase in $q_{e,\max}$ at pH of 13, respectively, Table 3. To elaborate this observation, we carried out the adsorption of CR on GO/PAMAM in a 0.1 M NaCl solution at 318 K. The result showed that the increase in ionic strength increased the binding constants and the adsorption capacities in region I and sections IIA and IIB of adsorption process when compared to those in water at 318 K, Figure 7 and Table 1, Table 2 and Table 3. This was due to the interaction of Na^+^ ions to ionic –NH_3_^+^ and polar –OH groups of adsorbent that increased their polarity. Similarly, the presence of Na^+^ ions increased the polarity of –NH_2_ groups of adsorbent at pH of 13, subsequently yielding higher CR binding constants and $q_{e,\max}$. It is noteworthy that, in a certain CR concentration range, the adsorption of CR molecules was leveled off at the end of section IIA at pHs of 10, 11, and 12, due to the presence of the repulsion interaction between negatively charged CR-adsorbed surface and free CR molecules, adsorption did not occur in a certain CR concentration range, as shown in Table 2. This adsorbate concentration range is called CRAC. CRAC is an abbreviation for “*concentration range of leveling off between two successive adsorption isotherm curves*”.

### 3.2. Adsorption Kinetic Modeling

**Figure 9:**
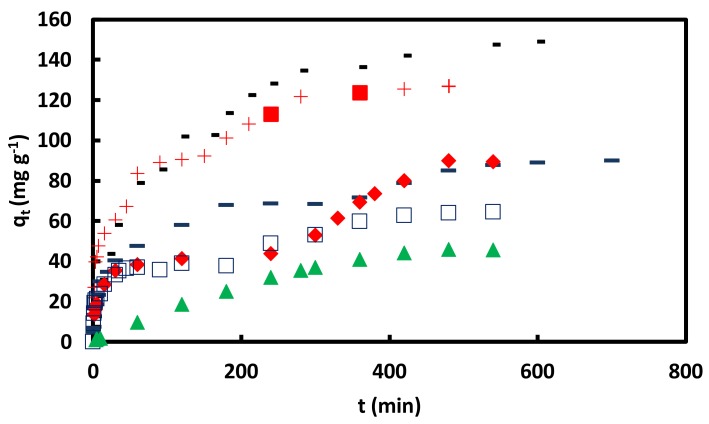
$q_{t}$ versus *t* for adsorption of CR on GO/PAMAM nanocomposite in + water, − 0.1 M NaCl, ▬ pH = 10, ♦ pH = 11, □ pH = 12 and ▲ pH = 13 solutions in 0.05 mM CR and at 318 K and 100 rpm.

**Figure 10:**
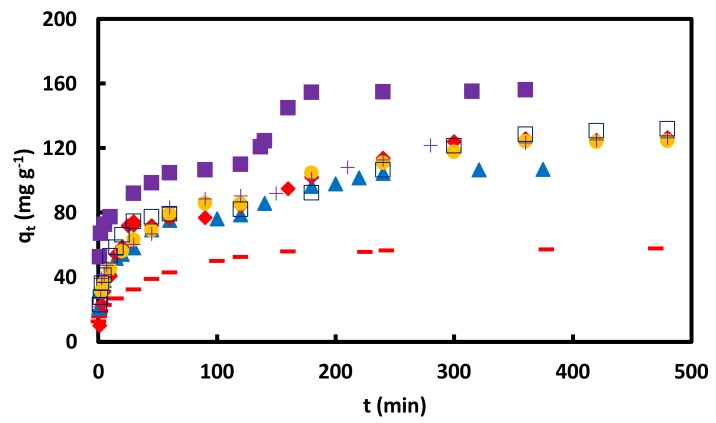
$q_{t}$ versus *t* for adsorption of CR on GO/PAMAM nanocomposite in ▬ 0.02, ▲ 0.05 and ■ 0.09 mM CR at 308 K and 100 rpm; in 0.05 mM CR at ♦ 40, ● 70 and +100 rpm at 318 K and □ 0.05 mM CR at 328 K and 100 rpm.

The adsorption kinetic experiments were performed in CR initial concentrations of 0.02, 0.05, and 0.09 mM, shaking rates of 40, 70, and 100 rpm, 0.1 M NaCl and alkaline solutions (pHs of 10, 11, 12, and 13) solutions, Figure 9 and Figure 10. Except in the CR initial concentration of 0.02 mM and the pH of 13 solutions, two or three successive curves were observed during the adsorption kinetic experiments. The obtained data were analyzed by the KASRA model and pore-diffusion and ISO equations, Table 5, Table 6, Table 7, Table 8, Table 9 and Table 10.

1. At the initial CR concentration ([*CR*]_0_) of 0.02 mM, which was in the section IIA of the ARIAN model, CR molecules interacted to stronger adsorption –NH_3_^+^ sites, and only one adsorption kinetic curve was observed, Table 6 and Table 7.
2. In [*CR*]_0_ = 0.05 and 0.09 mM (in section IIB of the ARIAN model) at different temperatures and shaking rates in water and 0.1 M NaCl and alkaline solutions (except for pH of 13) CR molecules interacted to –NH_3_^+^ sites, followed by interacting to –OH adsorption sites, and thus there were observed two adsorption kinetic curves, Table 6 and Table 7.
3. The adsorbent surface became negatively charged when CR molecules were adsorbed on –NH_3_^+^ (first available) sites of adsorbent in water and 0.1 M NaCl, and the hydroxyl and amine groups of the adsorbent were neutralized at pHs of 10, 11, 12, and 13. Thus, the diffusion of CR molecules into –OH sites of GO sheet of adsorbent was slowed down due to the repulsion interaction between free CR molecules and adsorbent surface, and the spatial hindrance of the adsorbed CR molecules. This “hampered” adsorption on –OH sites (the second curve) started after about 60-min TRAK intervals in water (except in [*CR*]_0_ = 0.02 mM) and 0.1 M NaCl solutions, Table 6 and Table 7.
4. In each experiment condition (CR concentration, temperature, shaking rate, ionic strength, and pH values) the adsorption acceleration, initial velocity, and $k_{dif}$ values of adsorption process decreased from region 1 to region 2 of the first kinetic curve, which was due to a decrease in CR concentration with increase in time. The values of these parameters in region 1 at 308 K were larger than those at other temperatures. To interpret these observations, two events should be considered. It seems that a higher temperature promotes the adsorption of CR on the adsorbent. On the other hand, the adsorption of CR on the adsorbent surface, as shown by zeta potential measurements, speeds up the agglomeration of adsorbent particles. The result of these two effects decreases initial kinetic parameters in region 1 at 318 and 328 K. Kinetics of adsorption may be controlled via film diffusion or intraparticle diffusion steps. In well-shaken adsorption systems, it is expected that film diffusion (external mass transfer resistance) in the aqueous phase is negligible [52]. As seen in Table 6 and Table 7, the increase in the shaking rate speeded up acceleration, $k_{dif}$ and initial velocities of adsorption process, which showed that in region 1 of the first kinetic curve, the adsorption of CR onto the adsorbent surface was the main rate-controlling step.
5. In region 2 of the first curve, $k_{dif}$ and initial velocity values of adsorption process decreased with the raise of shaking rate. This was due to an increase in the initial velocity of adsorption process, and thus a decrease in the CR concentration with the increase in shaking rate in region 1. For example, the CR concentration at *ssr* point, was 4.11 × 10^−5^ (after 5 min), 3.89 × 10^−5^ (after 5 min) and 3.79 × 10^−5^ (after 3 min) in 40, 70, and 100 rpm, respectively. On the other hand, the adsorption acceleration increased with the raise of shaking rate. These observations showed in region 2 of the first curve like its region 1, the interaction of CR with GO/PAMAM surface sites was the main rate-controlling step. It is concluded from results 4 and 5 that the –NH_3_^+^ sites are on the surface of adsorbent, Table 6 and Table 7.
6. In the single region of the second curve, adsorption acceleration (in the most cases), initial velocity, and $k_{dif}$ values were approximately constant with the increases in shaking rate, temperature, and CR concentration. It showed that the diffusion of CR molecules into the adsorbent particles (intraparticle diffusion) was the main rate-controlling step.As seen in Figure S1, H3 hysteresis loop of GO/PAMAM showed that nanocomposite structure is porous, and the BET isotherm showed that most of the adsorbent surface was covered by PAMAM. Furthermore, the SEM Image of GO/PAMAM at pH of 12 (Figure 3c) showed that the change of adsorbent surface due to repulsion interaction between neutralized ionizable –OH groups that were located inside (in pores) GO/PAMAM. Therefore, in a single region of the second curve, CR molecules were adsorbed on –OH sites of GO sheets of adsorbent, which were located in the pores of adsorbent particles, Table 6 and Table 7.
7. When comparing adsorption processes in 0.1 M NaCl and water solutions, using [*CR*]_0_ = 0.05 mM, 100 rpm and 318 K, the results showed that the adsorption acceleration, initial velocity, and $k_{dif}$ values in 0.1 M NaCl were less than those in water. These values were similar to those observed in the solution of [*CR*]_0_ = 0.05 mM, 40 rpm, and 318 K in water. It was speculated that doubly-negative charged CR molecules were surrounded by Na^+^ ions atmosphere, resulting in the decrease of CR adsorption kinetic parameters in Table 6 and Table 7.
8. In [*CR*]_0_ = 0.05 mM alkaline solutions (pHs of 10, 11, 12, and 13), with the increase in pH, all ionizable –OH groups and a number of –NH_3_^+^ groups of adsorbent were neutralized. This yielded a decrease in the number of adsorption sites of adsorbent, as well as the decrease in $q_{t}$ and final $q_{e}$. Because of the repulsion interaction between CR molecules and adsorbent surface, another small time interval appeared between regions 1 and 2 of the first kinetic curve in the KASRA model. At pH of 13, due to the neutralization of all –NH_3_^+^ groups, the first curve disappeared and after a 10-min interval, the –NH_2_ site curve was only observed. As the pH of solution increased, the initial velocity and the adsorption acceleration decreased in regions 1 and 2 of the first curve, and increased in the single-region second curve. The $k_{dif}$ values of adsorption process in the alkaline solutions decreased with the increase of pH. However, the $k_{dif}$ values were smaller than those in water under the same conditions, [*CR*]_0_ = 0.05 mM, 100 rpm and 318 K, Table 6 and Table 7.
9. Starting delay time at pH of 13 (10 min) and the first TRAKs at pHs of 10, 11, and 12 (2–3 min) were not observed both in water, and 0.1 M NaCl solutions. In addition, the second TRAKs at alkaline pHs were bigger than in water, and 0.1 M NaCl solutions, shown in Table 7 and Table 9. This could be attributed to the negative charges of adsorbent surface resulted from neutralization of –OH and –NH_3_^+^ groups. The independence of TRAK intervals of adsorption process from experimental conditions showed that the second type sites (–OH sites) were in the pores and far from their apertures. Based on all of the experiments in water and 0.1 M NaCl solutions, the results showed that their $q_{03}$ ($q_{t}$ at the beginning of the second kinetic curve) and the contribution of CR molecules on electrostatic charge values of adsorbent surface were in the range of 82.4–110.1 mg·g^−1^ and 2.4 × 10^−4^–3.2 × 10^−4^ C·g^−1^, respectively. These negative charges, in addition to the negative charge of adsorbent surface (from zeta potential measurement), resulted in the 60-min TRAK intervals before starting the adsorption on the second adsorption sites in these solutions. On the other hand, due to the significant contribution of neutralized –OH and –NH_3_^+^ groups on electrostatic charge values of adsorbent surface, the second TRAK intervals in pHs of 10, 11, and 12 were about 120 and 180 min. There was just a 10-min interval before beginning the adsorption on the –NH_2_ site of adsorbent at pH of 13, Table 7 and Table 9.
10. From Table 6, the total number of amine sites as –NH_3_^+^ and –NH_2_ sites ($q_{03}$) in water and alkaline solutions at pHs of 10, 11, 12, and 13 are 92.3, 89.1, 90, 63.9, and 46 mg·g^−1^, respectively. At pH of 12, results from zeta potential measurements and SEM images showed that phenolic –OH and –NH_3_^+^ groups of adsorbent were neutralized. Consequently, these caused a great change in morphology of adsorbent and poor dispersion in water. In fact, the exposure of these neutralized groups and their successive interaction to –NH_3_^+^ groups of adsorbent resulted in a sudden decrease in the number of total available amine groups of adsorbent at pHs of 12 and 13.
11. In each constant CR concentration, pH, temperature, ionic strength, and shaking rate, the second-order rate constants, which were obtained from the ISO equation in the first kinetic curve (–NH_3_^+^ site), decreased from the first part to the second part (part 2a), $k_{I1}^{2A}>k_{I2a}^{2A}$, whereas that of its third part (part 2b), $k_{I2b}^{2A}$, was bigger than both $k_{I1}^{2A}$ and $k_{I2a}^{2A}$. The former was due to the braking effect, [43] resulted from the decrease of CR concentration in solutions, the increase negative charge of adsorbent surface, and the spatial hindrance of adsorbed CR molecules, and the latter was due to sudden ending interaction at *kat* point. In the second kinetic curve (–OH sites), there is no region 1 and in region 2 of this curve the ISO second-order rate constant, like the first kinetic curve, decreased from the part 2a to the part 2b, $k_{I2a}^{2H}<k_{I2b}^{2H}$. In alkaline solutions, an increase in pH increased the surface negative charges, yielding a decrease in the intraparticle diffusion of CR molecules, and thus $k_{I2a}^{2A}$ and $k_{I2b}^{2A}$ values, Table 8 and Table 9.
12. The $E_{act}$ values of different regions of the first adsorption kinetic curve were calculated using $k_{I1}^{2A}$, $k_{I2a}^{2A}$, and $k_{I2b}^{2A}$ values that were obtained from the ISO equation for the adsorption in [*CR*]_0_ = 0.05 mM and 100 rpm at 308, 318, and 328 K, Table 9 and Table 10. $E_{act}$ values of region 1 and parts 2a and 2b of region 2 of the first adsorption kinetic curve were 21.8, 42.6, and 36.6 kJ mol^−1^, respectively. As expected, in the first kinetic curve, $E_{act}$ value of the most active –NH_3_^+^ sites in region 1 is less than those of other –NH_3_^+^ sites of the parts 2a and 2b in region 2. $E_{act}$ values of the first kinetic curve were calculable because the interaction between CR molecules and the adsorbent surface was the rate-controlling step (from results 5 and 6). Also, $E_{act}$ values of parts 2a and 2b of the second kinetic curve were not calculable, due to the oscillation of $k_{I2a}^{2H}$ and $k_{I2b}^{2H}$ values with temperature. In this kinetic curve, the diffusion of CR molecules into the adsorbent particles was the rate-controlling step (from result 7) and there is no reaction rate constant.

### 3.3. Regeneration of Adsorbent

**Figure 11:**
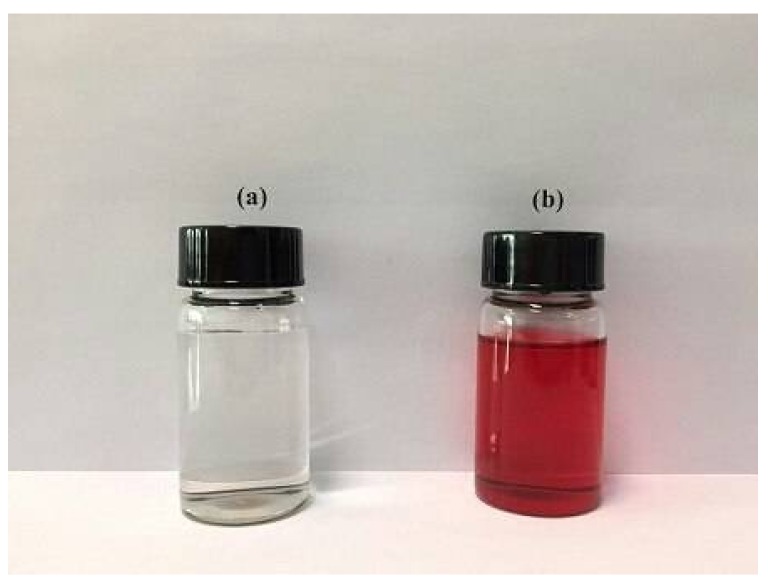
Ethylenediamine (**a**) before and (**b**) after extraction of CR from the CR-adsorbed GO/PAMAM.

Using acid and base to regenerate the CR-adsorbed GO/PAMAM was not possible. As shown before, acidic pHs produced more number of –NH_3_^+^ groups and at basic pHs approximately 30% of adsorption capacity of GO/PAMAM was occupied by adsorbed CR molecules. Also, solvents like ethanol, methanol, acetone, diethyl ether, and carbon tetrachloride could not extract adsorbed CR molecules from the CR-adsorbed GO/PAMAM. As shown in this work, parts of the adsorption sites of GO/PAMAM were amine groups. Thus, ethylenediamine as a solvent having amine groups was tested to regenerate the used GO/PAMAM. In a series of experiments, samples of 0.006 g GO/PAMAM in 15 mL of a 10^−4^ M CR solution were prepared. After completion of adsorption at 308 K and 100 rpm, the adsorbed CR molecules were extracted from the CR-adsorbed GO/PAMAM by adding 10 mL of ethylenediamine during four steps, Figure 11. This process took about 2 h. Adsorption capacities of the used adsorbent samples after the first and second regeneration cycles were 93–99% of its initial adsorption capacity, respectively.

On the other hand, two samples of GO/PAMAM after adsorption of CR at pH of 13 were regenerated and reused for adsorption of CR in water at 318 K and 100 rpm. As seen in Table 3, the adsorption capacity ($q_{e,\max}$) of GO/PAMAM for CR in pH of 13 was approximately 48% of its value in water. The adsorption capacities of this sample after the first and second regeneration cycles were about 51% and 98% of those of the adsorbent samples that totally had been used in neutral water, respectively.

**Figure 12:**
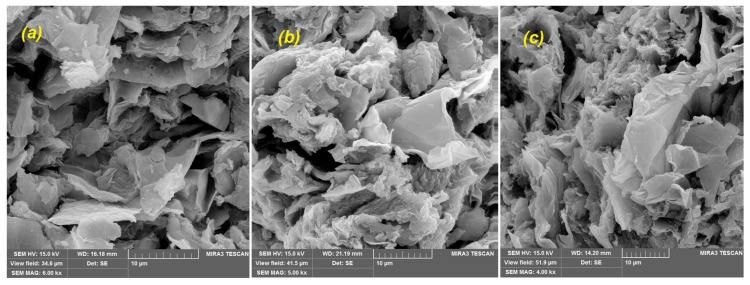
SEM images of CR-adsorbed GO/PAMAM prepared by (**a**) fresh GO/PAMAM and (**b**) and (**c**) two times regenerated GO/PAMAM samples in water under different initial conditions. Samples (**b**) and (**c**) had adsorbed CR molecules in water and at pH of 13 before regeneration cycles, respectively.

As shown in Table 2 and Table 3, at 318 K and 100 rpm, the contribution of amine groups in adsorption capacity of adsorbent at pH = 13 solution ($q_{e,\max}=81$ mg·g^−1^) was about 13% less than its value in water ($q_{\mathrm{sscB}}=93$ mg·g^−1^), and about 50% of total adsorption capacity of GO/PAMAM in water ($q_{e,\max}=167.6$ mg·g^−1^). This decrease in the number of adsorption sites supported our previous statement that changes observed in SEM images of GO/PAMAM in alkaline solutions in alkaline solutions was caused by the neutralization of phenolic hydroxyl groups that are located in pores of adsorbent and the repulsion interaction between them made the structure of adsorbent somehow inside out. The successive hydrogen binding between these neutralized groups with amine groups made a number of amine groups inactive too. However, results of regeneration of adsorbent showed that this change in the adsorbent structure was reversible. SEM images of the CR-adsorbed GO/PAMAM samples that were prepared by fresh GO/PAMAM and two times regenerated GO/PAMAM samples used in water (under different initial conditions) indicated that their surface characteristics were similar to each other, Figure 12.

Finally, the ethylenediamine that was used for the regeneration process was recycled by distillation process [53].

## 4. Conclusions

Adsorption kinetics and thermodynamics of doubly-negative charged CR molecules on the GO/PAMAM porous nanocomposite were studied at different pH, temperature, ionic strength, initial CR concentration, and shaking rate to elucidate the adsorption mechanism. Adsorption isotherms were analyzed by the ARIAN model and adsorption kinetic curves were studied by the KASRA model and the ISO and pore-diffusion equations. There were two kinds of adsorption sites on the adsorbent surface. The first type of adsorption sites were –NH_3_^+^ groups of PAMAM dendrimers, and the second ones were –OH groups of GO sheets of adsorbent. The –NH_3_^+^ sites were located on the surface and the –OH groups were placed in the pores of adsorbent. At the first step, CR molecules were adsorbed on the most active –NH_3_^+^ sites, followed by the adsorption to the less active –NH_3_^+^ sites. The adsorption of CR molecules on the –NH_3_^+^ sites made the adsorbent surface negatively charged, thus increasing the repulsion interaction between CR molecules and adsorbent. After the –NH_3_^+^ sites were fully occupied by CR molecules, the repulsion interaction resulted in a 60-min delay (in water) prior to the adsorption of CR molecules on –OH sites.

In alkaline solutions with pHs of 10, 11, and 12, all of the ionizable –OH groups and a portion of –NH_3_^+^ groups were neutralized. Hence, the –NH_3_^+^ and –NH_2_ groups were the first and second available CR adsorption sites. The adsorption of CR molecules on –NH_3_^+^ sites caused a high negative charge density on the adsorbent surface, subsequently yielding a 120-min interval before the adsorption of CR molecules on the –NH_2_ groups. Under the caustic environment, the neutralization of hydroxyl groups of the adsorbent increased the negative charges of the adsorbent surface. This caused the appearance of a small interval between the adsorption on the most active and less active –NH_3_^+^ groups of the adsorbent at the beginning of the adsorption. Equilibrium binding constants that were obtained from the Henry and Temkin equations at 308, 318, and 328 K, showed that the adsorption on the –NH_3_^+^ and –OH sites were endothermic, which supported the role of hydrophobic interactions in this process. Furthermore, the study of kinetics and thermodynamics of adsorption at alkaline solutions showed that the neutralization of –OH and –NH_3_^+^ groups of adsorbent decreased CR binding constants and the adsorption capacity of adsorbent.

In 0.1 M NaCl solution, when compared to water, adsorption kinetic parameters decreased and adsorption capacity increased. The former was resulted from surrounding CR molecules by Na^+^ ions, and the latter was the result of surrounding –NH_3_^+^ sites by Na^+^ ions. Adsorption kinetic parameters, i.e., $k_{dif}$, adsorption acceleration, and the initial velocity of adsorption indicated that the interaction of CR to the –NH_3_^+^ sites was the rate-controlling step. Based on the ISO equation, the $E_{act}$ values of CR adsorption on most active –NH_3_^+^ sites were less than those of less active –NH_3_^+^ sites, due to the braking effect. On the other hand, kinetic parameters showed that the intraparticle diffusion was the rate-controlling step during the interaction of CR molecules to –OH sites. Finally, the used adsorbent was fully regenerated by using ethylenediamine.

## Figures and Tables

**Table 1:** The $SSC_{A}$ and $q_{sscA}$ values and parameters that were obtained from the Henry’s law for the adsorption of CR on GO/PAMAM in water and 0.1 M NaCl solutions in region I at 308–328 K.

Solvent	T		Henry’s Law		$SSC_{A}$	$q_{sscA}$
	(K)	*A*	*K*	*R*^2^	(mM)	(mg·g^−1^)
Water	308	−0.461	4.08 × 10^7^	0.99	5.8 × 10^−4^	23.5
	318	−0.462	5.01 × 10^7^	0.98	5.4 × 10^−4^	25.1
	328	−0.745	6.20 × 10^7^	0.98	3.9 × 10^−4^	24.2
0.1 M NaCl	318	−0.365	5.39 × 10^7^	0.99	6.3 × 10^−4^	34.4
pH = 10	318	1.36	7.43 × 10^6^	0.98	3.3 × 10^−3^	24.7
pH = 11	318	0.17	6.61 × 10^6^	0.98	8.9 × 10^−3^	20.3
pH = 12	318	−0.34	2.57 × 10^6^	0.99	1.0 × 10^−3^	16.6
pH = 13	318	−0.22	3.74 × 10^6^	0.99	4.9 × 10^−3^	18.7

Units of *A* and *K* are in mg·g^−1^ and mg·g^−1^·M^−1^, respectively. Henry’s law for experimental data is as $q_{e}=Kc_{e}+A$.

**Table 2:** The $SSC_{B}$ and $q_{sscB}$ values and parameters that were obtained from the Temkin and Langmuir equations for the adsorption of CR on GO/PAMAM in water, 0.1 M NaCl, and alkaline solutions in section IIA at 308–328 K.

Solvent	T	Temkin			Langmuir			CRAC	$SSC_{B}$	$q_{sscB}$
	(K)	$c_{1}$	$c_{2}$	*R*^2^	$q_{mor}$	*K*	*R*^2^	(10^5^ M)	(mM)	(mg·g^−1^)
Water	308	19.09	5.71 × 10^6^	0.98	71.0	8.77 × 10^5^	0.99	-	3.9 × 10^−3^	62.1
	318	26.17	6.44 × 10^6^	0.97	98.0	1.07 × 10^6^	0.94	-	4.6 × 10^−3^	93.0
	328	25.78	6.80 × 10^6^	0.98	125.0	6.36 × 10^5^	0.97	-	6.0 × 10^−3^	96.3
0.1 M NaCl	318	53.1	2.99 × 10^6^	0.99	1158.7	4.95 × 10^4^	0.98	-	1.9 × 10^−3^	93.1
pH = 10	318	17.65	1.27 × 10^6^	0.99	76.9	1.44 × 10^5^	0.99	1.5–2.5	2.5 × 10^−2^	53.7
pH = 11	318	19.27	3.09 × 10^5^	0.98	83.3	3.54 × 10^4^	0.98	3.6–4.5	4.5 × 10^−2^	53.8
pH = 12	318	11.56	4.32 × 10^5^	0.99	49.8	5.06 × 10^4^	0.99	3.1–4.1	4.8 × 10^−2^	44.4
pH = 13	318	24.27	3.86 × 10^5^	0.99	102.0	4.49 × 10^4^	0.99	-	-	-

Unit of $q_{mor}$ and $c_{1}$ is in mg·g^−1^. Unit of $c_{2}$ and *K* is in M^−1^.

**Table 3:** The $c_{ma}$ and $q_{e,\max}$ values and parameters obtained from the Temkin and Langmuir equations for adsorption of CR on GO/PAMAM in section IIB in water, 0.1 M NaCl, and alkaline solutions at 308–328 K.

Solvent	T	Temkin			Langmuir			$c_{ma}$	$q_{e,\max}$
	(K)	$c_{1}$	$c_{2}$	*R*^2^	$q_{mor}$	*K*	*R^2^*	(mM)	(mg·g^−1^)
Water	308	51.88	8.61 × 10^5^	0.99	224.0	9.92 × 10^4^	0.99	2.5 × 10^−2^	155.9
	318	49.0	1.33 × 10^6^	0.97	211.0	1.70 × 10^5^	0.99	2.2 × 10^−2^	167.6
	328	47.3	1.49 × 10^6^	0.97	240.0	1.16 × 10^5^	0.97	2.6 × 10^−2^	160.2
0.1 M NaCl	318	53.4	2.95 × 10^6^	0.99	233.0	3.46 × 10^5^	0.99	1.3 × 10^−2^	198.0
pH = 10	318	29.43	2.77 × 10^5^	0.97	142.9	2.33 × 10^4^	0.97	7.2 × 10^−2^	85.9
pH = 11	318	80.55	3.57 × 10^4^	0.98	−268.8	−3074	0.98	7.7 × 10^−2^	80.1
pH = 12	318	71.8	3.88 × 10^4^	0.98	−275.5	−29,510	0.97	7.9 × 10^−2^	77.8
pH = 13	318	-	-	-	-	-	-	7.6 × 10^−2^	81.0

Unit of $q_{mor}$ and $c_{1}$ is in mg·g^−1^. Unit of $c_{2}$ and *K* is in M^−1^.

**Table 4:** Relative magnitude of region I and sections IIA and IIB for adsorption of CR on GO/PAMAM in water, 0.1 M NaCl, and alkaline solutions at 308–328 K.

Solvent	T (K)	First Region	Section IIA	Section IIB
Water	308	0.15	0.25	0.60
	318	0.15	0.41	0.44
	328	0.15	0.45	0.40
0.1 M NaCl	318	0.17	0.30	0.53
pH = 10	318	0.29	0.33	0.38
pH = 11	318	0.21	0.37	0.42
pH = 12	318	0.19	0.23	0.58
pH = 13	318	0.39	0.61	-

**Table 5:** Coefficients of the KASRA equation for kinetics of CR adsorption on GO/PAMAM nanocomposite at different temperatures and in various shaking rates and initial CR concentrations.

Solvent	T	[*CR*]_0_	rpm	KASRA Region 1 (1st Curve)				KASRA Region 2 (1st Curve)				KASRA Single Region (2nd Curve)			
	(K)	(mM)		*A*	*B*	*C*	*R*^2^	*A*	*B*	*C*	*R*^2^	*A*	*B*	*C*	*R*^2^
**Corresponding to:**				**ARIAN Region I (–NH_3_^+^ Site)**				**ARIAN Section IIA (–NH_3_^+^ Site)**				**ARIAN Section IIB (–OH Site)**			
Water	308	0.02	100	−17.51	34.35	0	0.99	−0.002	0.515	19.65	0.99	-	-	-	-
	308	0.05	100	−18.78	51.16	0	0.98	−0.008	1.150		0.98	−8 × 10^−4^	0.48	34.68	0.98
	308	0.09	100	−19.29	72.25	0	0.99	−0.007	1.055	66.81	0.99	−0.001	1.35	−26.71	0.98
	318	0.05	40	−0.815	10.21	0.27	0.98	−0.005	2.13	20.60	0.98	−7 × 10^−4^	0.50	33.47	0.98
	318	0.05	70	−1.60	15.33	0	0.98	−0.008	1.26	32.50	0.98	−5 × 10^−4^	0.38	48.26	0.98
	318	0.05	100	−2.35	19.48	1.20	0.96	−0.012	1.185	37.62	0.97	−4 × 10^−4^	0.41	41.17	0.99
	328	0.05	100	−3.62	22.32	1.10	0.97	−0.03	2.41	30.32	0.99	−1 × 10^−4^	0.25	52.40	0.99
0.1 M NaCl	318	0.05	100	−0.58	7.35	0.59	0.98	−0.005	1.26	19.69	0.98	−7 × 10^−4^	0.56	34.89	0.96
**Corresponding to:**				**ARIAN Region I (–NH_3_^+^ Site)**				**ARIAN Section IIA (–NH_3_^+^ Site)**				**ARIAN Section IIB (–NH_2_ Site)**			
pH = 10 *	318	0.05	100	−4.78	17.86	−0.20	0.99	−0.011	1.21	15.46	0.97	−3 × 10^−4^	0.36	–20.02	0.99
pH = 11 *	318	0.05	100	−5.16	18.31	0	0.97	−0.010	1.11	13.47	0.99	−8 × 10^−5^	0.26	–14.6	0.99
pH = 12 *	318	0.05	100	−4.29	0.19	0.25	0.99	−0.008	0.79	17.50	0.99	−3 × 10^−4^	0.265	–0.56	0.99
pH = 13	318	0.05	100	$q_{t}=0$ mg g^−1^ at t=0–4 min and $q_{t}≅1.5$ mg g^−1^ at t=4–10 min				-	-	-	-	−2 × 10^−4^	0.17	0.29	0.96

Units of *A*, *B,* and *C* are in mg·g^−1^ min^−2^, mg·g^−1^·min^−1^ and mg·g^−1^, respectively.* There are two interval time ranges between two successive adsorption kinetic curves (TRAKs) between regions 1 and 2 of the first kinetic curve and between the first and second kinetic curves (Table 7 and Table 9).

**Table 6:** Experimental $t_{e}$, $q_{e}$, $t_{02}$, $q_{02}$, $t_{03}$ and $q_{03}$ values and coefficients of the KASRA equation for kinetics of CR adsorption on GO/PAMAM at different temperatures and in various shaking rates and initial CR concentrations.

Solvent	T	[*CR*]_0_	rpm	$\left(t_{e},q_{e}\right)$	KASRA Regions 1 and 2 (1st Curve)					KASRA Single Region (2nd Curve)		
	(K)	(mM)			$a_{1}$	$v_{01}$	$\left(t_{02},q_{02}\right)$	$a_{2}$	$v_{02}$	$\left(t_{03},q_{03}\right)$	$a_{3}$	$v_{03}$
			**Corresponding to:**		**ARIAN Region I and Section IIA (–NH_3_^+^ Site)**					**ARIAN Section IIB (–OH Site)**		
Water	308	0.02	100	(160, 56.2)	−35.02	34.35	(2, 19.5)	−0.004	0.51	-	-	-
	308	0.05	100	(240, 104.6)	−37.56	51.16	(1, 32.4)	−0.016	1.18	(140, 86.1)	−0.002	0.255
	308	0.09	100	(180, 154.8)	−38.58	72.25	(2, 67.3)	−0.014	1.03	(120, 110.1)	−0.002	1.11
	318	0.05	40	(360, 126.0)	−1.63	10.21	(5, 31.1)	−0.010	2.09	(120, 84.1)	−1.4 × 10^−4^	0.33
	318	0.05	70	(360, 124.3)	−3.20	15.33	(5, 36.7)	−0.016	1.18	(120, 85.8)	−0.001	0.26
	318	0.05	100	(360, 123.6)	−4.70	19.48	(3, 39.7)	−0.024	1.11	(150, 92.3)	−0.001	0.29
	328	0.05	100	(360, 128.9)	−7.24	22.32	(3, 36.6)	−0.060	2.23	(120, 82.4)	−2 × 10^−4^	0.23
0.1M NaCl	318	0.05	100	(420, 142.2)	−1.16	7.35	(5, 23.0)	−0.010	1.21	(160, 102.8)	−1.4 × 10^−4^	0.56
			**Corresponding to:**		**ARIAN Region I and Section IIA (–NH_3_^+^** **Site)**					**ARIAN Section IIB (–NH_2_ Site)**		
pH = 10 *	318	0.05	100	(600, 89.1)	−9.16	17.86	(3, 17.1)	−0.02	1.14	(360, 71.7)	−6.1 × 10^−4^	0.15
pH = 11 *	318	0.05	100	(480, 90.0)	−10.32	18.31	(3, 16.1)	−0.020	1.05	(240, 44.0)	−1.6 × 10^−4^	0.22
pH = 12 *	318	0.05	100	(480, 63, 9)	−8.56	0.19	(5, 21.3)	−0.017	0.71	(180, 37.8)	−5.4 × 10^−4^	0.17
pH = 13	318	0.05	100	(480, 46.0)	0	0	(4, 1.5)	0	0	(10, 1.9)	−4 × 10^−4^	0.16

Units of $a_{1}$, $a_{2}$ and $a_{3}$ are in mg g^−1^ min^−2^ and those of $v_{01}$, $v_{02}$ and $v_{03}$ are in mg g^−1^·min^−1^. Units of $t_{e}$, $t_{02}$ and $t_{03}$ are in min and those of $q_{e}$, $q_{02}$ and $q_{03}$ are in mg·g^−1^. In region 1, $t_{01}$ and $q_{01}$ are equal to zero.* There are two TRAKs between regions 1 and 2 of the first kinetic curve and between the first and second kinetic curves (Table 7 and Table 9).

**Table 7:** Coefficients of the pore-diffusion equation for kinetics of CR adsorption on GO/PAMAM nanocomposite at different temperatures and in various shaking rates and initial CR concentrations.

Solvent	T	[*CR*]_0_	rpm	KASRA Regions 1 and 2 (1st Curve)					KASRA Single Region (2nd Curve)			TRAK
	(K)	(mM)		$k_{dif}$	*I*	$\left(t_{2},q_{2}\right)$	$k_{dif}$	*I*	$\left(t_{3},q_{3}\right)$	$k_{dif}$	*I*	(min)
		**Corresponding to:**		**ARIAN Region I and Section IIA (–NH_3_^+^ Site)**					**ARIAN Section IIB (–OH Site)**			
Water	308	0.02	100	18.3	−0.02	(0.8, 15.6)	3.6	13.8	-	-	-	-
	308	0.05	100	31.9	−6.8	(1, 32.4)	6.4	25.3	(140, 86.1)	5.0	28.0	60–120
	308	0.09	100	34.7	18.3	(2, 67.3)	5.9	59.2	(120, 110.1)	4.0	41.5	60–120
	318	0.05	40	14.1	−1.4	(5, 31.1)	12.2	1.5	(120, 84.1)	6.3	16.0	25–90
	318	0.05	70	16.9	0.44	(5, 36.7)	7.5	21.3	(120, 105)	3.2	61.2	90–120
	318	0.05	100	18.9	0.12	(5, 42.2)	6.2	29.1	(150, 92.3)	6.4	14.3	60–120
	328	0.05	100	20.7	−0.21	(3, 36.6)	10.3	19.6	(120, 82.4)	6.1	13.4	45–120
0.1M NaCl	318	0.05	100	7.3	−0.1	(1, 7.4)	9.4	1.3	(120, 102.0)	6.2	31.1	120–160
		**Corresponding to:**		**ARIAN Region I and Section IIA (–NH_3_^+^ Site)**					**ARIAN Section IIB (–NH_2_ Site)**			
pH = 10 *	318	0.05	100	12.75	−0.85	(3, 17.1)	5.0	11.0	(360, 71.7)	3.8	−0.3	180–360
pH = 11 *	318	0.05	100	11.6	0.34	(3, 16.1)	5.3	7.2	(240, 44.0)	7.4	−72.2	120–240
pH = 12 *	318	0.05	100	13.8	−0.05	(5, 21.3)	3.5	13.7	(180, 37.8)	3.5	−7.4	45–180
pH = 13	318	0.05	100	0	0	-	0	0	(10, 1.9)	2.6	−8.2	-

Units of $k_{dif}$ and *I* are in mg·g^−1^·min^−0.5^, mg·g^−1^ and min^−0.5^. Units of $t_{1}$, $t_{2}$ and $t_{3}$ are in min and those of $q_{1}$, $q_{2}$ and $q_{3}$ are in mg·g^−1^ and $t_{1}=q_{1}=0$. Boundary points coordinates in diffusion regions, $\left(t_{n},q_{n}\right)$, are similar to those of the KASRA model, $\left(t_{0n},q_{0n}\right)$ in Table 6. * There are two TRAKs between regions 1 and 2 of the first kinetic curve and between the first and second kinetic curves (Table 7 and Table 9).

**Table 8:** Coefficients of region 1 and region 2 (parts 2a and 2b) of the ISO equation for kinetics of CR adsorption on the –NH_3_^+^ (first available) sites of GO/PAMAM (on the first kinetic curve) at 308–328 K.

Solvent	T	[*CR*]_0_	rpm	$k_{I1}^{2A}$	$\left(t_{ssr}^{A},q_{ssr}^{A}\right)$	$k_{I2a}^{2A}$	$\left(t_{sp}^{A},q_{sp}^{A}\right)$	$k_{I2b}^{2A}$	$\left({\left[CR\right]}_{T}^{2},t_{T}^{2},q_{T}^{2}\right)$
	(K)	(mM)	Corresponding to ARIAN Region I and Section IIA (–NH_3_^+^ Site)						
Water	308	0.02	100	2322	(5, 23.0)	2063	(60, 43.2)	4642	(3.9 × 10^−3^, 160, 56.2)
	308	0.05	100	1550	(5, 38.3)	848	(30, 58.5)	2155	(2.8 × 10^−2^, 60, 75.6)
	308	0.09	100	1402	(5, 73.0)	534	(30, 92.2)	768	(6.0 × 10^−2^, 60, 105.0)
	318	0.05	40	2352	(5, 31.1)	1500	(15, 54.1)	3170	(2.9 × 10^−2^, 25, 72.0)
	318	0.05	70	1277	(10, 45.4)	639	(45, 69.2)	2315	(2.5 × 10^−2^, 90, 85.9)
	318	0.05	100	2486	(7, 47.7)	389	(45, 67.3)	3419	(2.6 × 10^−2^, 90, 89.0)
	328	0.05	100	2594	(5, 41.4)	2406	(20, 66.6)	5150	(2.7 × 10^−2^, 45, 77.6)
0.1 M NaCl	318	0.05	100	1661	(5, 23.0)	779	(60, 79.0)	260	(1.9 × 10^−2^, 120, 102)
* pH = 10	318	0.05	100	Table 9	(3, 17.1)	421	(30, 40.5)	263	(3.8 × 10^−2^, 180, 67.9)
* pH = 11	318	0.05	100	Table 9	(3, 16.1)	1053	(30, 35.6)	605	(3.8 × 10^−2^, 120, 41.5)
* pH = 12	318	0.05	100	Table 9	(5, 21.3)	1778	(30, 33.4)	3557	(3.9 × 10^−2^, 45, 36.6)
pH = 13	318	0.05	100	0	-	0	-	0	(4.9 × 10^−2^, 10, 1.9)

Unit of $k_{I1}^{2A}$, $k_{I2a}^{2A}$ and $k_{I2b}^{2A}$ is in M^−1^·min^−1^ and that of $t_{ssr}^{A}$, $t_{sp}^{A}$ and $,t_{T}^{2}$ is in min. Unit of $q_{ssr}^{A}$, $q_{sp}^{A}$ and $q_{T}^{2}$ is in mg·g^−1^. Units of [*CR*]_0_ and [*CR*]_T_ is in mM. ${\left[CR\right]}_{T}^{2}$, $t_{T}^{2}$ and $q_{T}^{2}$ are CR concentration, time and adsorption capacity at the beginning of the TRAK between region 2 of the first kinetic curve and second kinetic curve, respectively (corresponding to ${\left[CR\right]}_{e}$, $t_{e}$ and $q_{e}$ in the last curve). Superscript of *A* refers to –NH_3_^+^ site. $k_{I1}^{2A}$, $k_{I2a}^{2A}$, and $k_{I2b}^{2A}$ are the rate constants of adsorption on –NH_3_^+^ site in region 1 and region 2 (parts 2a and 2b) of the ISO equation, respectively. * Data of region 1 of the ISO equation have been given in Table 9.

**Table 9:** Coefficients of region 1 of the ISO equation for kinetics of CR adsorption on the –NH_3_^+^ (first available) sites of GO/PAMAM (on the first kinetic curve) at different pH values (from Table 8).

Solvent	T	[*CR*]_0_	rpm	$k_{I1}^{2A}$	$\left({\left[CR\right]}_{T}^{1},t_{T}^{1},q_{T}^{1}\right)$	TRAK	$\left(t_{ssr}^{A},q_{ssr}^{A}\right)$
	(K)	(mM)	Corresponding to ARIAN Region I (–NH_3_^+^ Site)				
pH = 10	318	0.05	100	4.9 × 10^4^	(3.8 × 10^−2^, 2, 17.1)	2–3	(3, 17.1)
pH = 11	318	0.05	100	1.6 × 10^5^	(4.5 × 10^−2^, 2, 16)	2–3	(3, 16.1)
pH = 12	318	0.05	100	1.1 × 10^5^	(4.5 × 10^−2^, 2, 19.4)	2–5	(5, 21.3)

Unit of $k_{I1}^{2A}$ is in M^−1^·min^−1^ and that of $t_{ssr}^{A}$ and $t_{T}^{1}$ is in min. Unit of $q_{ssr}^{A}$ and $q_{T}^{1}$ is in mg·g^−1^. Unit of [*CR*]_0_ and ${\left[CR\right]}_{T}^{1}$ is in mM. ${\left[CR\right]}_{T}^{1}$, $t_{T}^{1}$ and $q_{T}^{1}$ are CR concentration, time and adsorption capacity in the beginning of the TRAK between regions 1 and 2 of the first kinetic curve, respectively. Superscript of *A* refers to –NH_3_^+^ site.

**Table 10:** Coefficients of region 2 (part 2a and 2b) of the ISO equation for kinetics of CR adsorption on the hydroxyl or –NH_2_ sites of GO/PAMAM (on the second kinetic curve) at 308–328 K.

**Solvent**	**T**	**[*CR*]_0_**	**rpm**	$\left(t_{ssr}^{H},q_{ssr}^{H}\right)$	$k_{I2a}^{2H}$	$\left(t_{sp}^{H},q_{sp}^{H}\right)$	$k_{I2b}^{2H}$	$\left({\left[CR\right]}_{e},t_{e},q_{e}\right)$
	**(K)**	**(mM)**		**Corresponding to ARIAN Section IIB (–OH Site)**				
Water	308	0.02	100	-	-	-	-	-
	308	0.05	100	(120, 78.8)	758	(200, 98.0)	2221	(1.9 × 10^−2^, 240, 104.6)
	308	0.09	100	(120, 110.1)	230	(140, 124.7)	1255	(4.8 × 10^−2^, 180, 154.8)
	318	0.05	40	(120, 84.1)	616	(240, 113.8)	5890	(1.5 × 10^−2^, 360, 126.0)
	318	0.05	70	(120, 85.8)	480	(240, 111.0)	686	(1.5 × 10^−2^, 360, 124.3)
	318	0.05	100	(150, 92.3)	307	(240, 113.1)	493	(1.6 × 10^−2^, 360, 123.6)
	328	0.05	100	(120, 82.4)	154	(240, 106.8)	1231	(1.3 × 10^−2^, 360, 128.9)
0.1M NaCl	318	0.05	100	(100, 102.8)	327	(210, 122.6)	980	(9.2 × 10^−3^, 420, 142.2)
**Solvent**	**T**	**[*CR*]_0_**	**rpm**	$\left(t_{ssr}^{NH},q_{ssr}^{NH}\right)$	$k_{I2a}^{2NH}$	$\left(t_{sp}^{NH},q_{sp}^{NH}\right)$	$k_{I2b}^{2NH}$	$\left({\left[CR\right]}_{e},t_{e},q_{e}\right)$
	**(K)**	**(mM)**		**Corresponding to ARIAN Section IIB (–NH_2_ Site)**				
pH = 10	318	0.05	100	(360, 71.7)	346	(480, 85.1)	692	(2.89 × 10^−2^, 600, 89.1)
pH = 11	318	0.05	100	(240, 44.0)	324	(420, 80.1)	407	(2.2 × 10^−2^, 480, 90.0)
pH = 12	318	0.05	100	(180, 37.8)	275	(360, 59.9)	744	(3.2 × 10^−2^, 480, 63.9)
pH = 13	318	0.05	100	(10, 19)	109	(240, 32.2)	190	(3.7 × 10^−2^, 480, 46.0)

Unit of $k_{I2a}^{2H}$ and $k_{I2b}^{2H}$ is in M^−1^·min^−1^ and that of $t_{ssr}^{H}$, $t_{sp}^{H}$ and $t_{e}$ is in min. Unit of $q_{ssr}^{H}$, $q_{sp}^{H}$ and $q_{e}$ is in mg·g^−1^. Unit of $k_{I2a}^{2NH}$ and $k_{I2b}^{2NH}$ is in M^−1^·min^−1^ and that of $t_{ssr}^{NH}$, $q_{sp}^{NH}$, and $t_{e}$ is in min. Unit of $q_{ssr}^{NH}$, $q_{sp}^{NH}$, and $q_{e}$ is in mg·g^−1^. Unit of [*CR*]_0_ and [*CR*]_e_ is in mM and [*CR*]_e_ is CR concentration at *kat* point. The *H* and *NH* superscripts refer to hydroxyl and –NH_2_ sites (on the second kinetic curve), respectively.

## Supplementary Materials

The following are available online at http://www.mdpi.com/1996-1944/11/4/496/s1, Figure S1: BET diagram of GO/PAMAM.
