# Supplementary material for: Study of Adsorption Mechanism of Congo Red on Graphene Oxide/PAMAM Nanocomposite
Source: Materials (Basel). 2018 Mar 26;11(4):496. doi: 10.3390/ma11040496 (PMC5951342; doi:10.3390/ma11040496)
Supplement: Supplementary file 1 [file materials-11-00496-s001.pdf]

materials

MDPI

Supplementary

Study of Adsorption Mechanism of Congo Red on Graphene Oxide/PAMAM Nanocomposite

Mohammad Rafi, Babak Samiey and Chil-Hung Cheng

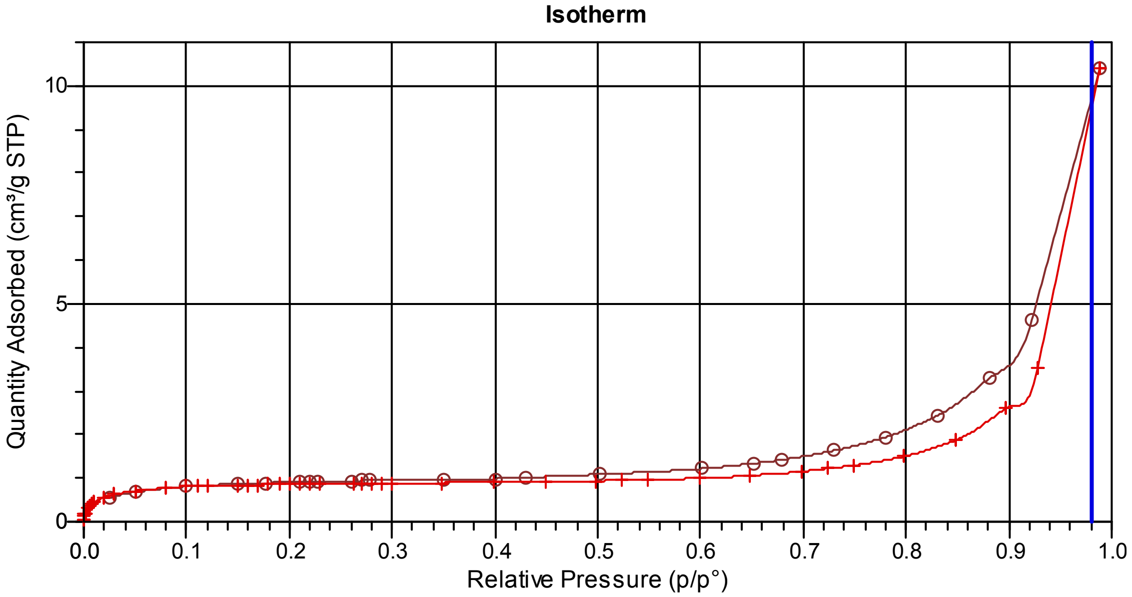

Figure S1. BET diagram of GO/PAMAM.
